# Interaction between a MAPT variant causing frontotemporal dementia and mutant APP affects axonal transport

**DOI:** 10.1016/j.neurobiolaging.2018.03.033

**Published:** 2018-08

**Authors:** Robert Adalbert, Stefan Milde, Claire Durrant, Kunie Ando, Virginie Stygelbout, Zehra Yilmaz, Stacey Gould, Jean-Pierre Brion, Michael P. Coleman

**Affiliations:** a Signalling Programme, The Babraham Institute, Babraham Research Campus, Cambridge, UK; b John van Geest Centre for Brain Repair, Department of Clinical Neurosciences, University of Cambridge, Cambridge, UK; c Laboratory of Histology, Neuroanatomy and Neuropathology, Faculty of Medicine, ULB Neuroscience Institute, Université Libre de Bruxelles, Brussells, Belgium

**Keywords:** Axonal transport, Alzheimer's disease, FTDP-17T, P301L mutation, Aβ, Mitochondria

## Abstract

In Alzheimer's disease, many indicators point to a central role for poor axonal transport, but the potential for stimulating axonal transport to alleviate the disease remains largely untested. Previously, we reported enhanced anterograde axonal transport of mitochondria in 8- to 11-month-old MAPT^P301L^ knockin mice, a genetic model of frontotemporal dementia with parkinsonism-17T. In this study, we further characterized the axonal transport of mitochondria in younger MAPT^P301L^ mice crossed with the familial Alzheimer's disease model, TgCRND8, aiming to test whether boosting axonal transport in young TgCRND8 mice can alleviate axonal swelling. We successfully replicated the enhancement of anterograde axonal transport in young MAPT^P301L/P301L^ knockin animals. Surprisingly, we found that in the presence of the amyloid precursor protein mutations, MAPT^P301L/P3101L^ impaired anterograde axonal transport. The numbers of plaque-associated axonal swellings or amyloid plaques in TgCRND8 brains were unaltered. These findings suggest that amyloid-β promotes an action of mutant tau that impairs axonal transport. As amyloid-β levels increase with age even without amyloid precursor protein mutation, we suggest that this rise could contribute to age-related decline in frontotemporal dementia.

## 1 Introduction

Alzheimer's disease (AD) is the most common form of dementia and is characterized by the presence of amyloid plaques and neurofibrillary tangles in brain tissue (Masters et al., 2015). Many studies indicate that axonal transport defects play an important role in the pathogenesis of AD, and overexpression of tau, whose mutation causes some types of frontotemporal dementia, has also been reported to impair axonal transport (Bertrand et al., 2013, Ittner et al., 2008, Majid et al., 2014). Axonal transport is impaired in several amyloid and tau mouse models, and further impairment of transport increases pathology (Adalbert and Coleman, 2013, De Vos et al., 2008, Millecamps and Julien, 2013). These include transgenic mouse models overexpressing amyloid precursor protein (APP) or familial Alzheimer's disease (FAD) mutant APP (Salehi et al., 2006, Stokin et al., 2005), mutant presenilin-1 (Lazarov et al., 2007), and wild-type and frontotemporal dementia with parkinsonism (FTDP) mutant tau (Ishihara et al., 1999, Zhang et al., 2004). The mechanisms by which the pathologic forms of APP, tau, PS1, and amyloid-β (Aβ) disrupt axonal transport are not properly understood. Aβ itself and overexpression of either wild-type and FTDP tau disrupt axonal transport of a variety of cargoes including mitochondria (Hiruma et al., 2003, Rui et al., 2006, Stamer et al., 2002, Zhang et al., 2004). Live-imaging studies of axonal transport in nervous system tissue have largely focused on mitochondria (Gilley et al., 2012, Mar et al., 2014, Marinkovic et al., 2012, Milde et al., 2015, Misgeld et al., 2007). We have reported live imaging of peripheral nerve explants of mitochondrial transport in a MAPT^P301L/P301L^ knockin mutant mouse and in normal aging (Gilley et al., 2012, Milde et al., 2015).

Axonal swellings are a pathologic feature common to human AD and to mice expressing pathogenic forms of APP or tau and mice expressing ApoE4 (Lewis et al., 2000, Tesseur et al., 2000, Wirths et al., 2006). Our earlier study showed that large axonal swellings in one FAD model, TgCRND8 mice, precede axon loss by many months and that axonal transport may be disrupted specifically at these swellings (Adalbert et al., 2009). TgCRND8 mice inherit a highly aggressive amyloidopathy caused by the expression of double mutant amyloid precursor protein (APP; K670N/M671L plus V717F) (Chishti et al., 2001), leading to early dystrophic neurites and some cell death (Bellucci et al., 2007). These findings raise the question of whether boosting axonal transport can alleviate the pathology. Until now, very few methods have been available to enhance axonal transport in vivo, and no studies have addressed whether this type of intervention can alleviate amyloid pathology or the associated axonal damage. There have been encouraging results using microtubule (MT)-stabilizing agents, such as those used in the treatment of cancer, to improve axonal transport in tauopathy models (Brunden et al., 2012, Zhang et al., 2005). These drugs, however, elicit severe side effects, especially as they have to be administered on a chronic basis, including axonal damage in peripheral neuropathy (Boyette-Davis et al., 2013). In previous work, we have enhanced axonal transport of mitochondria in young mice without adverse effects by knocking in the P301L mutation of MAPT (Gilley et al., 2012), a mutation causing FTDP-17T in humans (Wolfe, 2009). The effect on axonal transport was reversed as animals aged, suggesting other changes during aging alter how axons react to mutant MAPT. The mice remained healthy during the normal life span, with no overt tau pathology, motor, behavioral, or memory deficits. The initial aim of this study, therefore, was to test whether boosting axonal transport in young TgCRND8 mice by crossing them to MAPT^P301L^ knockin mice could alleviate axonal swelling.

We found no alterations in the number of plaque-associated axonal swellings or the number of amyloid plaques in TgCRND8 brains when MAPT mutation was introduced. However, while we successfully confirmed the enhancement of axonal transport in young MAPT^P301L/P301L^ mice and observed a trend in MAPT^P301L/+^ mice more representative of FTDP-17T patient genetics, the mitochondrial transport in the peripheral nerve axons of TgCRND8 mice showed a significant decrease in the presence of MAPT^P301L/P301L^, similar to that found in older MAPT^P301L/P301L^ mice. This suggests increasing amyloid beta peptide, or other APP processing products reverses an action of mutant tau on axonal transport and may contribute to a similar switch observed during normal aging.

## 2 Material and methods

### 2.1 Animals

All animal work was approved by the Babraham Institute Animal Welfare and Ethical Review Body and UK Home Office and carried out in accordance with the Animals (Scientific Procedures) Act, 1986, under Project Licenses 80/2254 and 70/7620.

First to confirm whether the enhanced mitochondrial transport is found in 3-month-old homozygous MAPT^P301L^ knockin mice (MAPT^P301L/P301L^), knockin mice were crossed with Mito-P (Thy-1-mitoCFP-P) mice as previously described (Gilley et al., 2012, Misgeld et al., 2007). Mito-P mice were kindly provided by Prof. Martin Kerschensteiner (University Munich) and Prof. Thomas Misgeld (TU Munich). Heterozygous male TgCRND8 mice (Chishti et al., 2001) on a 50:50 C57BL/6:129Sv background were bred to double homozygous MAPT^P301L^ knockin/Mito-P or MAPT^P301L^ knockin/YFP-H (Thy1.2-YFP-H) (Feng et al., 2000) female mice to generate triple heterozygotes TgCRND8/MAPT^P301L^/Mito-P or TgCRND8/MAPT^P301L^/YFP-H mice. These triple heterozygotes were backcrossed to MAPT^P301L^ homozygotes to generate offspring that were heterozygous for mutant APP and Mito-P (or YFP-H) and homozygous for MAPT^P301L^. Those mice which were heterozygous for Mito-P were used for transport studies, whereas those with YFP-H were used to assess histopathology. Controls without MAPT^P301L^ knockin were generated by crossing to non-MAPT^P301L^ mice on the same genetic background. Animals were kept on a 12:12 hours light:dark cycle at a constant temperature of 19 °C in a pathogen-free environment with up to 5 animals per cage.

### 2.2 Live imaging of axonal transport and image analysis

Imaging of mitochondrial transport and analysis was performed as described previously (Gilley et al., 2012, Milde et al., 2015). Briefly, mitochondrial movements were imaged in sciatic nerve explants using an Olympus Cell^R^ imaging system (IX81 microscope, Hamamatsu ORCA ER camera, ×100 1.45 NA apochromat objective). Peripheral nerves were used as a surrogate for central nervous system (CNS) tissue because in our hands quantitative analysis of axonal transport here is more reproducible than in CNS (Milde et al., 2015), but the presence of Aβ in peripheral nerves was confirmed (Fig. 2). During imaging, tissues were maintained in oxygenated Neurobasal-A medium at 37 °C in an environment chamber (Solent Scientific Ltd). Images were captured using fixed light intensity and camera exposure time settings at a rate of 2 frames per second for 5 minutes. Five to 10 individual movies (often containing multiple axons) were captured for each tissue explant. Individual axons were straightened using the Straighten plugin in ImageJ software, version 1.44 (Rasband, W.S., ImageJ, US National Institutes of Health, Bethesda, MD, USA; http://imagej.nih.gov/ij/, 1997e2012). Axonal transport parameters were determined for individual axons using the Difference Tracker set of ImageJ plugins (Andrews et al., 2010). The principal output of these plugins is the number of moving particles identified in each frame of the image, normalized to axon length (presented as particle count per second per 100 μm axon length).

**Fig. 1:**
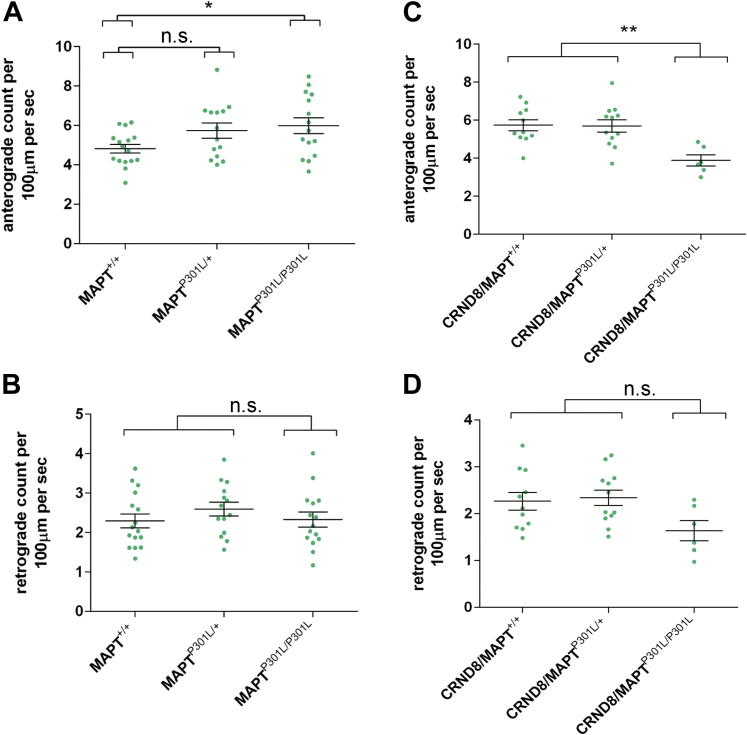
Changes in mitochondria transport in TgCRND8 mice homozygous for MAPT^P301L^ tau knockin mutation. Quantification of anterograde (A and C) and retrograde (B and D) mitochondria transport in sciatic nerves from 3-month-old mice with indicated genotypes (all mice are MitoP positive). For all graphs, each data point represents the mean value obtained for 1 animal (5 fields of view and, on average, 15 axons per animal). Horizontal bar indicates mean and error bars indicate standard error of the mean. Statistically significant differences between genotypes are indicated (*p < 0.05, **p < 0.01; 1-way analysis of variance with Tukey multiple comparisons posttest). Abbreviation: n.s., not significant.

**Fig. 2:**
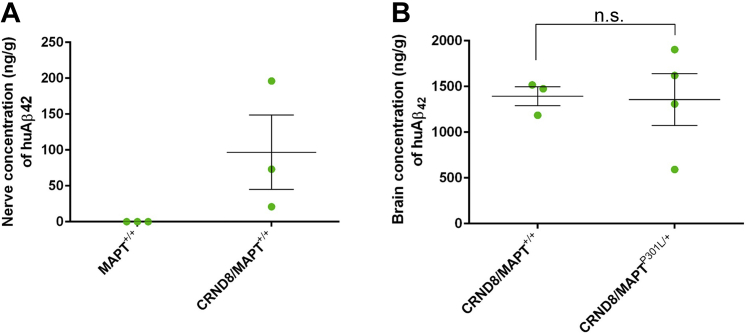
Human Aβ_1–42_ is present in peripheral nerves of TgCRND8 mice, and its levels in brain are independent of the MAPT^P301L^ knockin mutation. (A) Concentration of human Aβ_1–42_ (ng/g) in sciatic nerves of 3-month-old wild-type (MitoP) and TgCRND8 mice. (B) Brain concentration of human Aβ_1–42_ (ng/g) in TgCRND8 and TgCRND8 mice heterozygotes for the MAPT^P301L^ knockin mutation. Each data point represents 1 animal (mean and standard error of the mean, unpaired *t* test). Abbreviation: n.s., not significant.

### 2.3 Histology and staining

Mice were perfused transcardially with 4% phosphate-buffered paraformaldehyde, and brains and spinal cords processed as previously described (Adalbert et al., 2009). Sagittal brain sections were cut at 50 μm, and serial transverse spinal cord sections were cut at 25 μm through the L3–L5 segments using a Leica CM 1850 cryostat. To stain fibrillar amyloid deposits, brain sections were incubated in 0.02% Thioflavine S in tris-buffered saline (TBS) for 10 minutes and then rinsed in 50% ethanol in TBS and 100% TBS before mounting in Vectashield Mounting Medium. For motor neuron analysis, the spinal cord sections were stained with 0.5% cresyl violet (Adalbert et al., 2006). The excised YFP-H sciatic nerves were incubated in 1% Triton X-100 (Sigma) in 0.1M PBS for 10 minutes at room temperature, washed extensively in 0.1M PBS, and then mounted in Vectashield.

### 2.4 Microscopy and quantitative/qualitative analysis

The stained sections were imaged with an Olympus FV1000 confocal microscope imaging system using a 40× NA 1.3 or 63 × NA 1.4 oil immersion objective. Neuronal structures and amyloid plaques were scanned using a multitrack configuration with laser excitation (ex.) lines and emission (em.) filters as follows: Thioflavine S 405 nm ex. 475–525 nm em. and YFP 488 nm ex. 505–550 nm em. Single confocal slices or z-stacks at 0.4–0.5 mm steps were acquired to generate the data images. Conventional fluorescence imaging was carried out using an Olympus IX 81 inverted microscope coupled to an Olympus U-TV0.5XC digital camera system. Bright-field images of Nissl-stained sections were acquired using an Olympus BX41 upright microscope (40× objective) and MicroPublisher 3.3 camera (QImaging).

The number of amyloid plaques stained with Thioflavin S was determined in 6 sagittal sections (3 sections/hemisphere) as previously described (Adalbert et al., 2009). Amyloid plaques that had at least 1 YFP-containing axonal dystrophy (cutoff size of the dystrophy 5 μm) in the immediate vicinity were counted as plaques associated with axonal swellings. Cresyl violet–stained motor neurons with centrally placed nuclei and cytoplasm rich in rough endoplasmic reticulum were considered normal morphology. Every second section from L3–L5 spinal cord segments was qualitatively analyzed. YFP positive axons from sciatic and tibial nerves with no fragmentation or swellings along their length were classified as intact (Beirowski et al., 2004).

### 2.5 Aβ ELISA

To determine the levels of human Aβ_1–42_ in brain and sciatic nerve, samples were analyzed using a commercially available ELISA kit (Life Tech: KHB3441). Brain and sciatic nerves were homogenized in 5 M guanidine hydrochloride (8 mL per gram of tissue) supplemented with 1× Protease Inhibitor Cocktail (Roche) for 3–4 hours at room temperature. The sample was then frozen at −20 °C until use. Before running in the ELISA, the homogenate was diluted 1:50 in ice cold reaction buffer (Dulbecco's PBS +0.03 % Tween +5 % BSA supplemented with 1x Protease Inhibitor Cocktail) and centrifuged for 20 minutes at 4 °C at 16,000 × *g*. The supernatant was then diluted (in the commercial kit dilution buffer) before undergoing ELISA. Briefly, samples were incubated with Aβ detection antibody for 3 hours at RT then thoroughly washed. HRP-conjugated antibody was added to sample wells for 30 minutes. After another wash step, samples were incubated with stabilized chromogen for 30 minutes. The reaction was stopped using an acid-based stop solution and absorbance read at 450 nm using a PheraStar FS plate reader. Samples were run with a standard curve (4-parameter fit) to obtain a concentration readout. Final concentration is given as ng of Aβ per gram of tissue (Harwell and Coleman, 2016).

### 2.6 Immunohistochemistry and Gallyas staining

Mouse brains were fixed in 10% formalin for 24 hours before embedding in paraffin. Then, 7-μm-thick tissue sections were stained with the Gallyas silver staining method to identify neurofibrillary tangles as reported previously (Gilley et al., 2012, Kuninaka et al., 2015). They were examined with a Zeiss Axioplan microscope, and digital images were acquired using an AxioCam HRc camera.

The immunohistochemical labeling was performed using the ABC method. Sections were treated with 0.3% H_2_O_2_ to inhibit endogenous peroxidase and then blocked in 10% vol/vol normal horse serum in TBS (0.01 M Tris, 0.15 M NaCl, pH 7.4). After overnight incubation with diluted PHF-1 or AT8 antibodies (Innogenetics), sections were incubated with biotin-conjugated horse anti-mouse antibodies followed by ABC complex (Vector Laboratories, Burlingame, CA, USA). Peroxidase activity was developed using diaminobenzidine as chromogen.

### 2.7 Statistical analysis

Statistical tests, as described in the figure legends, were performed using Prism software (GraphPad Software Inc, La Jolla, CA, USA). A *p* value of >0.05 was considered not significant (ns), and *p < 0.05, **p < 0.01, and ***p < 0.001 was significant.

## 3 Results

### 3.1 Enhanced mitochondrial transport in 3-month-old MAPT^P301L/P301L^ knockin mouse peripheral nerves

First, we wanted to confirm whether the enhanced mitochondrial transport found previously in 8–11 months homozygous MAPT^P301L^ tau knockin mice (Gilley et al., 2012) was also present in younger, 3-month-old homozygous tau knockin mice, an age when TgCRND8 shows substantial axonal swellings and an increasing plaque density (Adalbert et al., 2009). To test this, we crossed MAPT^P301L^ knockin mice to Thy-1-mitoCFP-P (Mito-P) mice, which have CFP-labeled mitochondria in a subset of their neurons (Misgeld et al., 2007). The transport of CFP-labeled mitochondria was assessed in axons of explanted sciatic nerves from 3-month-old MAPT^P301L/P301L^, MAPT^P301L/+^, and MAPT^+/+^ mice. Peripheral nerves were used because we find quantitative analysis of axonal transport in nerves is more reproducible than that in CNS (Milde et al., 2015). We found that the number of CFP-labeled mitochondria moving in an anterograde direction was significantly higher in MAPT^P301L/P301L^ mice relative to wildtypes (Fig 1A). In MAPT^P301L/+^ mice, there was a trend toward increased mitochondrial transport, but the difference was not statistically significant. No significant differences were found for retrograde transport (Fig 1B), and changes in anterograde flux were not accompanied by significant alterations to maximum speed or average speed (not shown). Thus, axons from young 3-month-old MAPT^P301L/P301L^ knockin mice show an increase in anterograde mitochondrial flux, similar to older (8–11 months) mice, prior to the reversal of this effect we have previously observed in old age (Gilley et al., 2012).

### 3.2 In the presence of the TgCRND8 mutation, MAPT^P301L/P301L^ reduces the axonal transport of mitochondria

Next, we assessed whether the presence of MAPT^P301L^ knockin mutation, alters the mitochondrial transport in TgCRND8 peripheral axons. Instead of the expected enhancement, we observed a significant decrease in anterograde mitochondrial flux in 3-month-old TgCRND8 mice homozygous for the MAPT^P301L^ knockin mutation (TgCRND8/MAPT^P301L/P301L^) (Fig 1C), reminiscent of old MAPT^P301L/P301L^ mice without mutant APP (Gilley et al., 2012). Retrograde mitochondrial transport also declined, but this was not statistically significant in our sample (Fig 1D), and average speed of transport was unaltered (not shown). No significant differences in any transport parameters were identified for TgCRND8 mice heterozygous for the tau knockin mutation (TgCRND8/MAPT^P301L/+^). These data indicate that in the presence of the TgCRND8 mutation, the effect of MAPT^P301L/P301L^ on axonal transport of mitochondria in young animals is reversed, suggesting an interaction between Aβ, or other APP processing products, and mutant tau that resembles the effect of normal aging.

### 3.3 Aβ_1–42_ is present in peripheral nerves of TgCRND8 mice and Aβ_1–42_ level in brain is not altered by MAPT^P301L/+^

Using ELISA, we quantified the Aβ_1–42_ in sciatic nerves from young (3 months) TgCRND8 mice and found that Aβ_1–42_ is present in peripheral nerves, at the age when the axonal transport measurements were conducted (Fig. 2A). However, the levels were less than 10% of those in brain of the same animals (Fig. 2B). This suggests that the use of peripheral nerves as a surrogate for CNS tissue in this study could underestimate the effect of Aβ, or other APP processing products, in combination with tau in the CNS. Brain levels of Aβ were not altered by the presence of the MAPT^P301L/+^ knockin mutation (Fig. 2B).

### 3.4 MAPT^P301L/P301L^ knockin mutation does not reduce the number of plaques and the associated axonal swellings in TgCRND8 brains

**Fig. 3:**
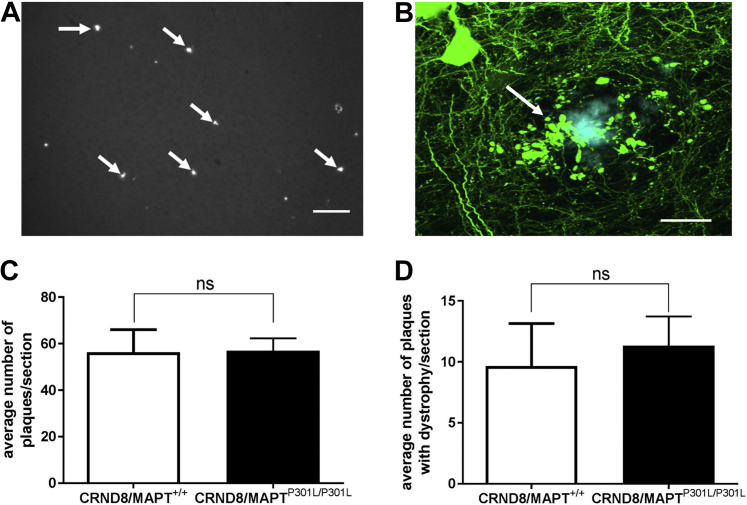
Homozygous MAPT^P301L^ knockin mutation does not reduce the number of plaques and the associated neurite swellings in TgCRND8 brains. (A) Amyloid plaques (arrows) in the cortex of 3-month-old TgCRND8 mouse and (B) a single amyloid plaque associated with neurite dystrophy (arrow). Quantification (mean and standard error of the mean, unpaired *t* test; n = 5) of the number of plaques (C), and plaques with associated dystrophy (D) reveals no significant difference (ns) between TgCRND8 and TgCRND8/MAPT^P301L/P301L^ mice. Blue: Thioflavin S; Green: YFP in B. Scale bar: A, 100 μm; B, 20 μm. (For interpretation of the references to color in this figure legend, the reader is referred to the Web version of this article.)

To test whether the presence of MAPT^P301L^ knockin mutation ameliorates amyloid-associated pathology, we quantified the number of amyloid plaques and the number of plaque-associated axonal swellings in the brains of TgCRND8/MAPT^+/+^/YFP-H and TgCRND8/MAPT^P301L/P301L^/YFP-H mice at 3 months of age. We found that the MAPT^P301L/P301L^ mutation did not significantly reduce the number of amyloid plaques (Fig 3A, C) or the associated axonal swellings in the brains of TgCRND8 mice (Fig 3B, D). Although as axonal transport was not enhanced in these animals as expected, it remains undetermined whether boosting transport can alleviate axonal swellings.

### 3.5 No morphologic alterations are present in TgCRND8/MAPT^P301L/P301L^ spinal cord motor neurons and peripheral nerves

**Fig. 4:**
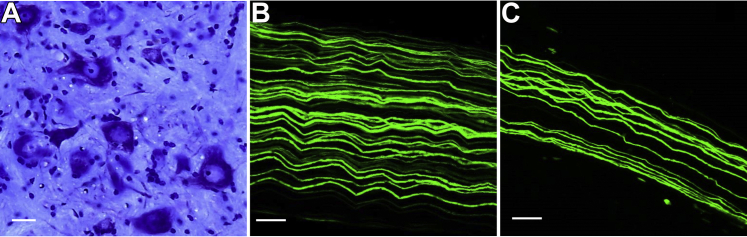
Morphologically intact spinal cord motor neurons and peripheral nerves in 3-month-old TgCRND8/MAPT^P301L/P301L^ mice. (A) Cresyl violet (Nissl)-stained transverse section through the L3-L5 lumbar spinal cord segment. (B) YFP positive axons from sciatic and (C) tibial nerves. Scale bar: A, 20 μm; B and C, 50 μm. (For interpretation of the references to color in this figure legend, the reader is referred to the Web version of this article.)

Histologic analysis of motor neurons from lumbar spinal cord segments L3-L5 and YFP positive axons from sciatic and tibial nerves showed no obvious alterations in 3-month-old TgCRND8/MAPT^P301L/P301L^ mice. Cresyl violet staining revealed motor neurons with normal morphology with centrally placed nuclei and a cytoplasm rich in rough endoplasmic reticulum (Fig 4A). Also, YFP positive axons from sciatic and tibial nerves were intact with no fragmentation or swellings along their length (Fig 4B, C). These observations show that the reduced anterograde axonal transport of mitochondria in peripheral nerves of TgCRND8/MAPT^P301L/P301L^ mice occurs in morphologically normal axons and spinal cord motor neurons.

### 3.6 Absence of tau pathology

**Fig. 5:**
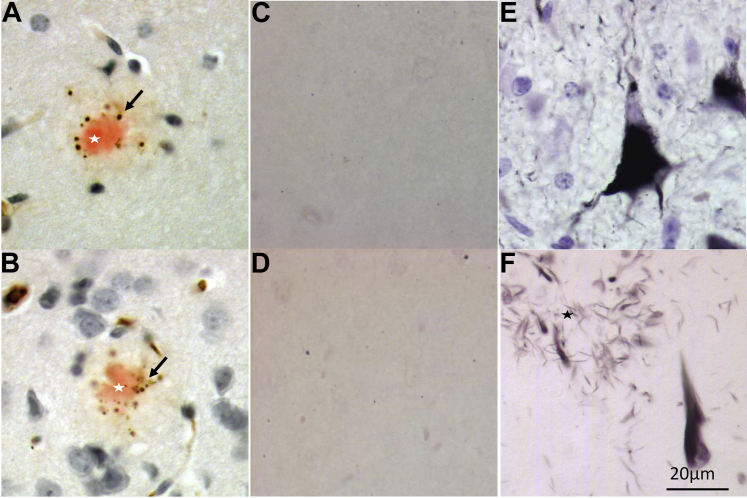
No tau histopathology in the brains of 6-month-old TgCRND8 mice homozygous for MAPT^P301L^ tau knockin mutation. (A, B) PHF1 immunostaining in the cortex showed only puncta-like staining of dystrophic neurites (arrow) around the congo red positive plaques (star) with no neuronal staining in the mouse frontal cortex of TgCRND8 (A) or TgCRND8/MAPT^P301L/P301L^ (B). (C–F) Gallyas silver staining. No NFTs was present in the mouse brains of TgCRND8 (C) or TgCRND8/MAPT^P301L/P301L^ (D). Tg30 tau transgenic mouse brain expressing human P301S/G272V mutant tau (Leroy et al., 2007) (E) or human AD tissue (F) acting as positive control for NFT and dystrophic neurites around amyloid plaque (star). Scale bars: 20 μm. (For interpretation of the references to color in this figure legend, the reader is referred to the Web version of this article.)

We previously showed that pathologic aggregation of hyperphosphorylated tau does not occur in MAPT^P301L/P301L^ tau knockin mice (Gilley et al., 2012). Similarly, in this study, no Gallyas-positive neurons or neuronal PHF-1 immunostaining for phosphorylated tau was detected in 6-month-old brains even after combining TgCRND8 transgene with MAPT^P301L/P301L^ or MAPT^P301L/+^ knockin mutation (Fig 5). Only a puncta-like staining of dystrophic neurites around the congo red positive plaques was found (Fig 5A). Hence, the presence of FAD-APP does not induce tau pathology in MAPT^P301L^ tau knockin mice, at least up to the age of 6 months.

## 4 Discussion

In this study, we confirmed and extended our previous observation of increased anterograde mitochondrial transport in 8- to 11-month-old homozygous MAPT^P301L^ knockin mice (Gilley et al., 2012) to 3-month-old homozygous knockin mice (Fig 1A) and to a trend in the same direction in heterozygotes. Retrograde transport (Fig 1B), maximum speed or average speed in either direction (not shown) were unaltered, and after crossing to TgCRND8, the numbers of plaque-associated axonal swellings or amyloid plaques in TgCRND8 brains were neither reduced nor increased by MAPT^P301L/P301L^.

By crossing the MAPT^P301L^ knockin mice with an FAD model, TgCRND8 mice, our initial aim was to test whether boosting axonal transport can alleviate axonal swellings. We have selected TgCRND8 mice for our study as they develop robust axonal swellings at an early stage in amyloid pathology that we have extensively characterized, preceding axon and cell death, and major loss of synapses (Adalbert et al., 2009). Although these features arise in young animals, they closely resemble those in an aging human AD brain. Therefore, it is reasonable to use this model to test whether they can be alleviated by boosting axonal transport. These axonal swellings contain accumulations of fragmented mitochondria and other organelles, which suggest impairment of axonal transport. Therefore, “unblocking” these swellings could alleviate AD-like histopathology and symptoms in TgCRND8 mice. Surprisingly, the presence of the homozygous MAPT^P301L^ mutation significantly decreased the anterograde mitochondrial flux in 3-month-old TgCRND8 mice instead of enhancing it as we initially hypothesized, so it remains untested at present whether increasing axonal transport could alleviate plaques and/or axonal swellings.

Previous studies indicated that the physiological function of tau is required for aspects of FAD-APP pathogenesis. The block of axonal transport by Aβ_1–42_ oligomers in hippocampal primary cultures requires the presence of tau (Vossel et al., 2010), and FAD-APP–induced behavioral deficits in mice are alleviated by removing tau (Roberson et al., 2007). Recently, it was also reported that mutant tau with markedly reduced MT-binding capacity still enabled Aβ-induced axonal transport deficits (Vossel et al., 2015). We now show that co-expression of MAPT^P301L^, even at physiological levels, synergistically impairs axonal transport with FAD-APP. All these studies suggest that different tau genotypes, in the presence of Aβ, can lead to transport impairment.

Our findings support the hypothesis that Aβ and tau converge in axons to influence axonal transport or that tau itself is a downstream target of Aβ on the same pathway. Previously, it was suggested that tau may lie downstream of APP/Aβ or on a necessary pathway that converges onto a common, downstream target (Karran et al., 2011, Morris et al., 2011). In different neurodegenerative diseases, both in humans and in mice, plaques and tangles can arise independently of one another. In mice, neither FAD-APP nor FTDP-17T mutant tau causes both of these pathological hallmarks. Some human patients show amyloid without tau pathology, and FTDP-17T tau mutations cause tangles without plaques in frontotemporal dementia. These observations are consistent with convergent disease pathways, either of which can cause pathology when sufficiently activated. MAPT^P301L^ knockin mutation does not enhance the generation of Aβ_1–42_ in the brains of 3-month-old TgCRND8 mice (Fig 2B), consistent with previous results using overexpression of tau in FAD-APP expressing cell lines or primary neuronal cultures (Goldsbury et al., 2006). In addition, when tau deletion reduced cognitive deficits and excitotoxic damage in FAD-APP mice, there was no corresponding reduction in Aβ_1–42_ (Roberson et al., 2007). This finding is important because if the synergistic effect of MAPT^P301L^ knockin tau and FAD-APP were mediated by increasing Aβ_1–42_ processing, then that would favor a mechanism involving feedback on APP over a common downstream target for Aβ_1–42_ and MAPT^P301L^ tau.

Tau pathology is not a feature of APP or PS1 mutant mice but has been seen when combined with overexpressing tau mutants. We were interested to see whether such pathology could be induced when mutant tau is expressed at physiological levels. The overexpression of Aβ did not induce pathologic aggregation of hyperphosphorylated tau in MAPT^P301L^ tau knockin mice by the age of 6 months; however, we cannot rule out the possibility that tau pathology could develop at an older age. The puncta-like staining of phosphorylated tau in dystrophic neurites around the plaques we observed in our study (Fig. 5A,B) was reported previously in mutant APP and APP/PS1 transgenic mice, therefore is unlikely to reflect an action of MAPT mutation (Boutajangout et al., 2004, Kurt et al., 2003, Tomidokoro et al., 2001).

It is worth noting that alternative models that overexpress both mutant APP and MAPT^P301L^ show late development of tau tangles; a double transgenic requiring 9 months and a different 3×Tg model not showing pathology until 12 months of age (Lewis et al., 2001, Oddo et al., 2003). It may be due to the knockin nature of the MAPT^P301L^ in our model that neurofibrillary pathology cannot develop during the normal life span of our mice.

Some evidence suggests that increased tau expression and/or altered tau isoform ratio are more important for the development of neurofibrillary pathology in a mouse than is the presence of an FTDP-17T mutation (Adams et al., 2009, Gotz et al., 2001). The presence of tau at physiological levels and with the expected isoform ratios in our MAPT^P301L^ tau knockin mice could be responsible for the absence of tau pathology. In addition, it could be that murine tau has less propensity for aggregation in vivo compared to human tau (Allen et al., 2002, Ando et al., 2011, Sahara et al., 2002).

The decline in axonal transport seen when combining the APP and MAPT^P301L^ mutations may anticipate the age-related effect of MAPT^P301L^ tau mutation alone. As Aβ levels increase with age even without APP mutation (Lesne et al., 2013), our double mutants may have modeled this process in younger animals. Thus, we suggest that this Aβ rise could contribute to age-related decline in frontotemporal dementia. It would be important to repeat our observations with other mutant tau knockin mice (e.g., R406W) as well, to know whether axonal transport impairment in the presence of Aβ is a consistent effect of FTDP tau mutations. In addition, it would be interesting to test whether amyloid-independent pathogenic mechanism can also lead to axonal transport impairment observed in our model, by crossing the MAPT^P301L^ tau knockin mice with wild-type human APP overexpressing mice (Mucke et al., 2000). In these mice, the human wild-type APP processing is basically nonamyloidogenic (Simon et al., 2009).

Using our mouse model, we found that Aβ_1–42_ is present in peripheral nerves at the age when the axonal transport measurements were done (Fig. 2A), similar to previous studies in the peripheral nerves of other APP mutant mice (Jolivalt et al., 2012). In our model, the Aβ_1–42_ levels in the peripheral nerves were less than 10% of those in the brains of the same animals, which suggest that the effect of Aβ in combination with tau in the CNS could be much stronger.

In the presence of the APP mutation, MAPT^P301L/P301L^ knockin failed to enhance axonal transport of mitochondria; therefore, new strategies have to be used to increase transport (Hinckelmann et al., 2013). Drugs increasing MT acetylation may be useful to boost axonal transport in AD. Tubulin acetylation is reduced in the brains of patients with AD (Hinckelmann et al., 2013) and in the brains of various mouse models of AD (Govindarajan et al., 2013, Kim et al., 2012). Studies so far showed that HDAC6 inhibitors reverse the amyloid beta–induced changes in α-tubulin acetylation and mitochondrial trafficking in cultured hippocampal neurons (Kim et al., 2012) and HDAC6 knockout in a mouse model of AD restores α-tubulin acetylation in the brain and cognitive function in AD mice (Govindarajan et al., 2013). Another approach to improve axonal transport could be to inhibit cJun N-terminal kinase (JNK) activity. JNK hyperactivity has been reported in models of AD, and the inhibition of JNK activity in vivo was shown to be neuroprotective in animal models (Mehan et al., 2011). JNK3 inhibition rescued axonal transport defects induced by pathogenic fragments of huntingtin in a squid model of axonal transport (Morfini et al., 2009).

In summary, we confirmed and extended our previous observation of an increase in mitochondrial transport in young MAPT^P301L^ knockin homozygous mice to animals aged 3 months and observed a nonsignificant trend also in heterozygotes. This could represent early consequences of the tau dysfunction that may be relevant to FTDP-17T pathogenesis in humans as they age. Our results demonstrate that TgCRND8 and MAPT^P301L/P301L^ mutations in combination reduce the axonal transport of mitochondria in peripheral nerves, in contrast to the enhancement of transport in young MAPT^P301L/P301L^ mice. This exciting finding suggests that Aβ and tau converge in axons to influence axonal transport or that tau itself is a downstream target of amyloid beta on the same pathway. MAPT^P301L^ mutation did not significantly reduce the number of plaque associated axonal swellings or the number of amyloid plaques in TgCRND8 brains and did not alter Aβ levels in brain. Our FAD-APP and MAPT^P301L^ tau knockin mouse model may be useful to study how amyloid beta peptide and tau combine in pathogenesis.

## Disclosure statement

The authors have no actual or potential conflicts of interest.

## References

[bib1] Adalbert R., Coleman M.P. (2013). Review: axon pathology in age-related neurodegenerative disorders. Neuropathol. Appl. Neurobiol..

[bib2] Adalbert R., Nogradi A., Babetto E., Janeckova L., Walker S.A., Kerschensteiner M., Misgeld T., Coleman M.P. (2009). Severely dystrophic axons at amyloid plaques remain continuous and connected to viable cell bodies. Brain.

[bib3] Adalbert R., Nogradi A., Szabo A., Coleman M.P. (2006). The slow Wallerian degeneration gene in vivo protects motor axons but not their cell bodies after avulsion and neonatal axotomy. Eur. J. Neurosci..

[bib4] Adams S.J., Crook R.J., Deture M., Randle S.J., Innes A.E., Yu X.Z., Lin W.L., Dugger B.N., McBride M., Hutton M., Dickson D.W., McGowan E. (2009). Overexpression of wild-type murine tau results in progressive tauopathy and neurodegeneration. Am. J. Pathol..

[bib5] Allen B., Ingram E., Takao M., Smith M.J., Jakes R., Virdee K., Yoshida H., Holzer M., Craxton M., Emson P.C., Atzori C., Migheli A., Crowther R.A., Ghetti B., Spillantini M.G., Goedert M. (2002). Abundant tau filaments and nonapoptotic neurodegeneration in transgenic mice expressing human P301S tau protein. J. Neurosci..

[bib6] Ando K., Leroy K., Heraud C., Yilmaz Z., Authelet M., Suain V., De Decker R., Brion J.P. (2011). Accelerated human mutant tau aggregation by knocking out murine tau in a transgenic mouse model. Am. J. Pathol..

[bib7] Andrews S., Gilley J., Coleman M.P. (2010). Difference tracker: ImageJ plugins for fully automated analysis of multiple axonal transport parameters. J. Neurosci. Methods.

[bib8] Beirowski B., Berek L., Adalbert R., Wagner D., Grumme D.S., Addicks K., Ribchester R.R., Coleman M.P. (2004). Quantitative and qualitative analysis of Wallerian degeneration using restricted axonal labelling in YFP-H mice. J. Neurosci. Methods.

[bib9] Bellucci A., Rosi M.C., Grossi C., Fiorentini A., Luccarini I., Casamenti F. (2007). Abnormal processing of tau in the brain of aged TgCRND8 mice. Neurobiol. Dis..

[bib10] Bertrand A., Khan U., Hoang D.M., Novikov D.S., Krishnamurthy P., Rajamohamed Sait H.B., Little B.W., Sigurdsson E.M., Wadghiri Y.Z. (2013). Non-invasive, in vivo monitoring of neuronal transport impairment in a mouse model of tauopathy using MEMRI. Neuroimage.

[bib11] Boutajangout A., Authelet M., Blanchard V., Touchet N., Tremp G., Pradier L., Brion J.P. (2004). Characterisation of cytoskeletal abnormalities in mice transgenic for wild-type human tau and familial Alzheimer's disease mutants of APP and presenilin-1. Neurobiol. Dis..

[bib12] Boyette-Davis J.A., Cata J.P., Driver L.C., Novy D.M., Bruel B.M., Mooring D.L., Wendelschafer-Crabb G., Kennedy W.R., Dougherty P.M. (2013). Persistent chemoneuropathy in patients receiving the plant alkaloids paclitaxel and vincristine. Cancer Chemother. Pharmacol..

[bib13] Brunden K.R., Ballatore C., Lee V.M., Smith A.B., Trojanowski J.Q. (2012). Brain-penetrant microtubule-stabilizing compounds as potential therapeutic agents for tauopathies. Biochem. Soc. Trans..

[bib14] Chishti M.A., Yang D.S., Janus C., Phinney A.L., Horne P., Pearson J., Strome R., Zuker N., Loukides J., French J., Turner S., Lozza G., Grilli M., Kunicki S., Morissette C., Paquette J., Gervais F., Bergeron C., Fraser P.E., Carlson G.A., George-Hyslop P.S., Westaway D. (2001). Early-onset amyloid deposition and cognitive deficits in transgenic mice expressing a double mutant form of amyloid precursor protein 695. J. Biol. Chem..

[bib15] De Vos K.J., Grierson A.J., Ackerley S., Miller C.C. (2008). Role of axonal transport in neurodegenerative diseases. Annu. Rev. Neurosci..

[bib16] Feng G., Mellor R.H., Bernstein M., Keller-Peck C., Nguyen Q.T., Wallace M., Nerbonne J.M., Lichtman J.W., Sanes J.R. (2000). Imaging neuronal subsets in transgenic mice expressing multiple spectral variants of GFP. Neuron.

[bib17] Gilley J., Seereeram A., Ando K., Mosely S., Andrews S., Kerschensteiner M., Misgeld T., Brion J.P., Anderton B., Hanger D.P., Coleman M.P. (2012). Age-dependent axonal transport and locomotor changes and tau hypophosphorylation in a “P301L” tau knockin mouse. Neurobiol. Aging.

[bib18] Goldsbury C., Mocanu M.M., Thies E., Kaether C., Haass C., Keller P., Biernat J., Mandelkow E., Mandelkow E.M. (2006). Inhibition of APP trafficking by tau protein does not increase the generation of amyloid-beta peptides. Traffic.

[bib19] Gotz J., Chen F., van Dorpe J., Nitsch R.M. (2001). Formation of neurofibrillary tangles in P301l tau transgenic mice induced by Abeta 42 fibrils. Science.

[bib20] Govindarajan N., Rao P., Burkhardt S., Sananbenesi F., Schluter O.M., Bradke F., Lu J., Fischer A. (2013). Reducing HDAC6 ameliorates cognitive deficits in a mouse model for Alzheimer's disease. EMBO Mol. Med..

[bib21] Harwell C.S., Coleman M.P. (2016). Synaptophysin depletion and intraneuronal Abeta in organotypic hippocampal slice cultures from huAPP transgenic mice. Mol. Neurodegener..

[bib22] Hinckelmann M.V., Zala D., Saudou F. (2013). Releasing the brake: restoring fast axonal transport in neurodegenerative disorders. Trends Cell Biol..

[bib23] Hiruma H., Katakura T., Takahashi S., Ichikawa T., Kawakami T. (2003). Glutamate and amyloid beta-protein rapidly inhibit fast axonal transport in cultured rat hippocampal neurons by different mechanisms. J. Neurosci..

[bib24] Ishihara T., Hong M., Zhang B., Nakagawa Y., Lee M.K., Trojanowski J.Q., Lee V.M. (1999). Age-dependent emergence and progression of a tauopathy in transgenic mice overexpressing the shortest human tau isoform. Neuron.

[bib25] Ittner L.M., Fath T., Ke Y.D., Bi M., van Eersel J., Li K.M., Gunning P., Gotz J. (2008). Parkinsonism and impaired axonal transport in a mouse model of frontotemporal dementia. Proc. Natl. Acad. Sci. U. S. A..

[bib26] Jolivalt C.G., Calcutt N.A., Masliah E. (2012). Similar pattern of peripheral neuropathy in mouse models of type 1 diabetes and Alzheimer's disease. Neuroscience.

[bib27] Karran E., Mercken M., De Strooper B. (2011). The amyloid cascade hypothesis for Alzheimer's disease: an appraisal for the development of therapeutics. Nat. Rev. Drug Discov..

[bib28] Kim C., Choi H., Jung E.S., Lee W., Oh S., Jeon N.L., Mook-Jung I. (2012). HDAC6 inhibitor blocks amyloid beta-induced impairment of mitochondrial transport in hippocampal neurons. PLoS One.

[bib29] Kuninaka N., Kawaguchi M., Ogawa M., Sato A., Arima K., Murayama S., Saito Y. (2015). Simplification of the modified gallyas method. Neuropathology.

[bib30] Kurt M.A., Davies D.C., Kidd M., Duff K., Howlett D.R. (2003). Hyperphosphorylated tau and paired helical filament-like structures in the brains of mice carrying mutant amyloid precursor protein and mutant presenilin-1 transgenes. Neurobiol. Dis..

[bib31] Lazarov O., Morfini G.A., Pigino G., Gadadhar A., Chen X., Robinson J., Ho H., Brady S.T., Sisodia S.S. (2007). Impairments in fast axonal transport and motor neuron deficits in transgenic mice expressing familial Alzheimer's disease-linked mutant presenilin 1. J. Neurosci..

[bib32] Leroy K., Bretteville A., Schindowski K., Gilissen E., Authelet M., De Decker R., Yilmaz Z., Buee L., Brion J.P. (2007). Early axonopathy preceding neurofibrillary tangles in mutant tau transgenic mice. Am. J. Pathol..

[bib33] Lesne S.E., Sherman M.A., Grant M., Kuskowski M., Schneider J.A., Bennett D.A., Ashe K.H. (2013). Brain amyloid-beta oligomers in ageing and Alzheimer's disease. Brain.

[bib34] Lewis J., Dickson D.W., Lin W.L., Chisholm L., Corral A., Jones G., Yen S.H., Sahara N., Skipper L., Yager D., Eckman C., Hardy J., Hutton M., McGowan E. (2001). Enhanced neurofibrillary degeneration in transgenic mice expressing mutant tau and APP. Science.

[bib35] Lewis J., McGowan E., Rockwood J., Melrose H., Nacharaju P., Van Slegtenhorst M., Gwinn-Hardy K., Paul Murphy M., Baker M., Yu X., Duff K., Hardy J., Corral A., Lin W.L., Yen S.H., Dickson D.W., Davies P., Hutton M. (2000). Neurofibrillary tangles, amyotrophy and progressive motor disturbance in mice expressing mutant (P301L) tau protein. Nat. Genet..

[bib36] Majid T., Ali Y.O., Venkitaramani D.V., Jang M.K., Lu H.C., Pautler R.G. (2014). In vivo axonal transport deficits in a mouse model of fronto-temporal dementia. Neuroimage. Clin..

[bib37] Mar F.M., Simoes A.R., Leite S., Morgado M.M., Santos T.E., Rodrigo I.S., Teixeira C.A., Misgeld T., Sousa M.M. (2014). CNS axons globally increase axonal transport after peripheral conditioning. J. Neurosci..

[bib38] Marinkovic P., Reuter M.S., Brill M.S., Godinho L., Kerschensteiner M., Misgeld T. (2012). Axonal transport deficits and degeneration can evolve independently in mouse models of amyotrophic lateral sclerosis. Proc. Natl. Acad. Sci. U. S. A..

[bib39] Masters C.L., Bateman R., Blennow K., Rowe C.C., Sperling R.A., Cummings J.L. (2015). Alzheimer's disease. Nat. Rev. Dis. Primers.

[bib40] Mehan S., Meena H., Sharma D., Sankhla R. (2011). JNK: a stress-activated protein kinase therapeutic strategies and involvement in Alzheimer's and various neurodegenerative abnormalities. J. Mol. Neurosci..

[bib41] Milde S., Adalbert R., Elaman M.H., Coleman M.P. (2015). Axonal transport declines with age in two distinct phases separated by a period of relative stability. Neurobiol. Aging.

[bib42] Millecamps S., Julien J.P. (2013). Axonal transport deficits and neurodegenerative diseases. Nat. Rev. Neurosci..

[bib43] Misgeld T., Kerschensteiner M., Bareyre F.M., Burgess R.W., Lichtman J.W. (2007). Imaging axonal transport of mitochondria in vivo. Nat. Methods.

[bib44] Morfini G.A., You Y.M., Pollema S.L., Kaminska A., Liu K., Yoshioka K., Bjorkblom B., Coffey E.T., Bagnato C., Han D., Huang C.F., Banker G., Pigino G., Brady S.T. (2009). Pathogenic huntingtin inhibits fast axonal transport by activating JNK3 and phosphorylating kinesin. Nat. Neurosci..

[bib45] Morris M., Maeda S., Vossel K., Mucke L. (2011). The many faces of tau. Neuron.

[bib46] Mucke L., Masliah E., Yu G.Q., Mallory M., Rockenstein E.M., Tatsuno G., Hu K., Kholodenko D., Johnson-Wood K., McConlogue L. (2000). High-level neuronal expression of abeta 1-42 in wild-type human amyloid protein precursor transgenic mice: synaptotoxicity without plaque formation. J. Neurosci..

[bib47] Oddo S., Caccamo A., Shepherd J.D., Murphy M.P., Golde T.E., Kayed R., Metherate R., Mattson M.P., Akbari Y., LaFerla F.M. (2003). Triple-transgenic model of Alzheimer's disease with plaques and tangles: intracellular Abeta and synaptic dysfunction. Neuron.

[bib48] Roberson E.D., Scearce-Levie K., Palop J.J., Yan F., Cheng I.H., Wu T., Gerstein H., Yu G.Q., Mucke L. (2007). Reducing endogenous tau ameliorates amyloid beta-induced deficits in an Alzheimer's disease mouse model. Science.

[bib49] Rui Y., Tiwari P., Xie Z., Zheng J.Q. (2006). Acute impairment of mitochondrial trafficking by beta-amyloid peptides in hippocampal neurons. J. Neurosci..

[bib50] Sahara N., Lewis J., DeTure M., McGowan E., Dickson D.W., Hutton M., Yen S.H. (2002). Assembly of tau in transgenic animals expressing P301L tau: alteration of phosphorylation and solubility. J. Neurochem..

[bib51] Salehi A., Delcroix J.D., Belichenko P.V., Zhan K., Wu C., Valletta J.S., Takimoto-Kimura R., Kleschevnikov A.M., Sambamurti K., Chung P.P., Xia W., Villar A., Campbell W.A., Kulnane L.S., Nixon R.A., Lamb B.T., Epstein C.J., Stokin G.B., Goldstein L.S., Mobley W.C. (2006). Increased App expression in a mouse model of Down's syndrome disrupts NGF transport and causes cholinergic neuron degeneration. Neuron.

[bib52] Simon A.M., Schiapparelli L., Salazar-Colocho P., Cuadrado-Tejedor M., Escribano L., Lopez de Maturana R., Del Rio J., Perez-Mediavilla A., Frechilla D. (2009). Overexpression of wild-type human APP in mice causes cognitive deficits and pathological features unrelated to Abeta levels. Neurobiol. Dis..

[bib53] Stamer K., Vogel R., Thies E., Mandelkow E., Mandelkow E.M. (2002). Tau blocks traffic of organelles, neurofilaments, and APP vesicles in neurons and enhances oxidative stress. J. Cell Biol..

[bib54] Stokin G.B., Lillo C., Falzone T.L., Brusch R.G., Rockenstein E., Mount S.L., Raman R., Davies P., Masliah E., Williams D.S., Goldstein L.S. (2005). Axonopathy and transport deficits early in the pathogenesis of Alzheimer's disease. Science.

[bib55] Tesseur I., Van Dorpe J., Bruynseels K., Bronfman F., Sciot R., Van Lommel A., Van Leuven F. (2000). Prominent axonopathy and disruption of axonal transport in transgenic mice expressing human apolipoprotein E4 in neurons of brain and spinal cord. Am. J. Pathol..

[bib56] Tomidokoro Y., Ishiguro K., Harigaya Y., Matsubara E., Ikeda M., Park J.M., Yasutake K., Kawarabayashi T., Okamoto K., Shoji M. (2001). Abeta amyloidosis induces the initial stage of tau accumulation in APP(Sw) mice. Neurosci. Lett..

[bib57] Vossel K.A., Xu J.C., Fomenko V., Miyamoto T., Suberbielle E., Knox J.A., Ho K., Kim D.H., Yu G.Q., Mucke L. (2015). Tau reduction prevents Abeta-induced axonal transport deficits by blocking activation of GSK3beta. J. Cell Biol..

[bib58] Vossel K.A., Zhang K., Brodbeck J., Daub A.C., Sharma P., Finkbeiner S., Cui B., Mucke L. (2010). Tau reduction prevents Abeta-induced defects in axonal transport. Science.

[bib59] Wirths O., Weis J., Szczygielski J., Multhaup G., Bayer T.A. (2006). Axonopathy in an APP/PS1 transgenic mouse model of Alzheimer's disease. Acta Neuropathol..

[bib60] Wolfe M.S. (2009). Tau mutations in neurodegenerative diseases. J. Biol. Chem..

[bib61] Zhang B., Higuchi M., Yoshiyama Y., Ishihara T., Forman M.S., Martinez D., Joyce S., Trojanowski J.Q., Lee V.M. (2004). Retarded axonal transport of R406W mutant tau in transgenic mice with a neurodegenerative tauopathy. J. Neurosci..

[bib62] Zhang B., Maiti A., Shively S., Lakhani F., McDonald-Jones G., Bruce J., Lee E.B., Xie S.X., Joyce S., Li C., Toleikis P.M., Lee V.M., Trojanowski J.Q. (2005). Microtubule-binding drugs offset tau sequestration by stabilizing microtubules and reversing fast axonal transport deficits in a tauopathy model. Proc. Natl. Acad. Sci. U. S. A..

